# The Osteocyte as a Novel Key Player in Understanding Periodontitis Through its Expression of RANKL and Sclerostin: a Review

**DOI:** 10.1007/s11914-019-00509-x

**Published:** 2019-03-28

**Authors:** Teun J. de Vries, Carmen Huesa

**Affiliations:** 1 Department of Periodontology, Academic Centre for Dentistry Amsterdam, University of Amsterdam and Vrije Universiteit Amsterdam, Gustav Mahlerlaan 3004, 1081 LA Amsterdam, The Netherlands; 2 Centre for Reproductive Health, Queen Margaret Research Institute, University of Edinburgh, Edinburgh, Little France Crescent, EH16 4TJ UK

**Keywords:** Osteocytes, Periodontitis, Alveolar bone, Osteoclasts, RANKL, Sclerostin

## Abstract

**Purpose of Review:**

Periodontitis is the inflammation-associated bone loss disease of the alveolar bone that surrounds teeth. Classically, the emphasis on the etiology of periodontitis has been on the products of periodontal pathogens that lead to an inflammatory response of the *soft* tissues of the periodontium, eventually leading to activation of osteoclasts that degrade the alveolar bone. Until recently, the response of osteocytes that populate the alveolar bone, and that are known for their regulatory role in bone anabolism and catabolism, has not been addressed.

**Recent Findings:**

This review demonstrates that osteocytes play a key contributing role in periodontitis progression in various experimental mouse and rat periodontitis models. Osteocytes are the key expressing cells of both osteoclast differentiation factor RANKL as well as osteoblast activity regulator sclerostin. Targeted deletion of RANKL in osteocytes prevents osteoclast formation, thereby impairing periodontitis, despite the pressure of periodontitis-associated bacteria. Antibodies against the osteocyte-derived protein sclerostin inhibit and partially revert periodontitis by stimulating bone formation.

**Summary:**

Experimental mouse and rat periodontitis models strongly indicate a key role for the bone-encapsulated osteocyte in understanding periodontitis etiology.

## Introduction

Recent literature has challenged the classical way periodontitis progression is considered, attributing a key role to the osteocyte. Thus far, the emphasis of this inflamed gums disease leading to bone loss has always been on the bacterial pressure activating the inflammatory compartment, which stimulates the activation of osteoclasts that ultimately leads to bone loss [1••, 2•]. This review will modify this view by including the recently elucidated role of RANKL and sclerostin expressing osteocytes in the pathogenesis of periodontitis.

### Of Mice and Men: a Brief Introduction into Periodontitis

Periodontitis, the most common bone-erosive disease, with prevalence in the USA of 46% among adults [3], is an inflammation-associated disease affecting the tissue that surrounds teeth, the periodontium. It is caused by a deviant dental bacterial biofilm, ultimately leading to the disease-characteristic recruitment of osteoclasts and their bone resorbing activity. It is generally considered a complex, multifactorial disease where lifestyle factors, such as smoking and dietary habits, and importantly genetic susceptibility, play a role in its progression and severity [4]. The use of in particular mouse models for studying the etiology of periodontitis helps to scale-down this complexity. Mice do not smoke, can be held on a standardized diet, and inbred strains are available, thereby circumventing individual genetic differences such as apparent between humans. Typical mouse experimental periodontitis approaches make use of a standardized exposure of the gums to periodontitis-associated bacteria such as *Aggregatibacter actinomycetemcomitans* (*Aa*), *Porphyromonas gingivalis* (*Pg*), or *Fusobacterium nucleatum* (*Fn*), or bacterial products such as lipopolysaccharide (LPS). By making use of knockout and transgenic approaches, mouse models are extremely useful in identifying genes that protect against periodontitis [2•]. In a recent approach unraveling mouse susceptibility to periodontitis, a genome-wide association study was applied to no less than 104 mouse strains with differing susceptibility to periodontitis. This identified the Cxcl family as periodontitis susceptibility genes [5].

### Cellular Players of Periodontitis Progression: the “Old View” Without Considering Osteocytes

Mouse studies were valuable in discerning the histological changes that take place during the progression of the disease. Mice that were infected with periodontitis-associated bacterium *Aa* showed progressive bone loss, concomitant with the first influx of neutrophils [6], followed by a subsequent T helper 1 and 2 (Th1 and Th2) cellular influx, together with Th17 cells and at the end of the chronic infection regulatory T cells (Tregs) [1••]. Studies in human tissues have confirmed such a sequence of influxes of inflammatory cells. When comparing gingivitis, which is considered as the pre-stage of periodontitis without bone loss to the more advanced stage periodontitis, it became apparent that more macrophages and more plasma cells populate periodontitis lesions [7]. Typical for periodontitis is the increase in infiltrated connective tissue (ICT) area, the area that is occupied by immune cells to the expense of fibroblasts and connective fibers. This area contains more Th17-cells [8] that secrete IL-17, a cytokine that is typical for advanced periodontitis [6] and that plays a role in activating osteoclasts [9]. The infiltrated T and B cells have been reported to express receptor activator of nuclear factor kappa B ligand (RANKL), which is considered as the key differentiation factor for osteoclast differentiation [10]. Experimental evidence also showed that the fibroblasts of the periodontium respond to periodontitis-associated bacteria by producing large quantities of inflammatory cytokines [11] and they play a role in leukocyte retention and survival [12], as well as in osteoclast formation [13].

Together, this inflammatory scene within the soft connective tissue of the periodontium sets the stage for activation of osteoclast precursors, where inflammatory cytokines such as IL-1β [14] and TNF-α [15] will enhance osteoclast formation and activity. However, this view does not take into account the recent appraisal of the osteocyte as a major RANKL producing cell [16, 17]. RANKL is the key osteoclast differentiation factor discovered some 20 years ago [18] that has boosted the field of osteoclast biology. Also, the classical view of histological changes during periodontitis does not consider the osteocyte as producer of the bone formation inhibitory protein sclerostin that inhibits the Wnt-signaling pathway [19, 20]. Below, the recently discovered role of osteocytes as cellular players in periodontitis progression is discussed, highlighting both the RANKL and the sclerostin pathway.

### RANKL Expressing Osteocytes and Periodontitis

**Fig. 1:**
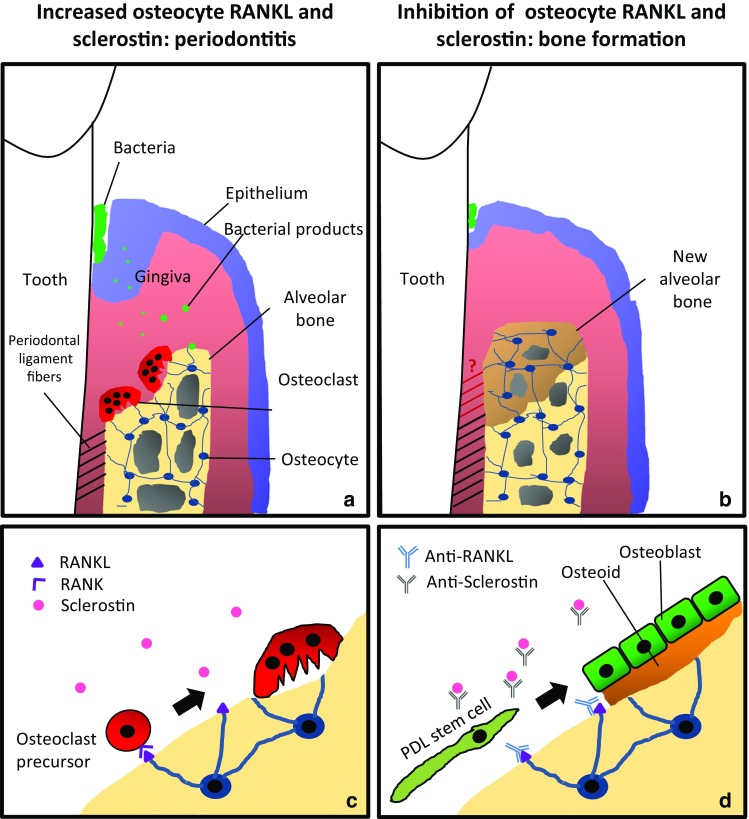
Role of osteocyte produced RANKL and sclerostin in periodontitis. This cartoon, based on experimental periodontitis studies in rats and mice, emphasizes the role of osteocytes in periodontitis progression. For simplification purposes, this schematic is devoid of immune cells that invade the periodontium and that also play an indispensable role for understanding the etiology of periodontitis. **a**, **c** Sclerostin [22] and bacterial products such as LPS [23] may increase osteocyte RANKL expression. RANKL produced by osteocytes could be a main contributor of periodontitis progression [24••]. **b**, **d** Inhibition of osteocyte RANKL and sclerostin induces bone formation [25, 26••, 27•, 28], which leads to increased alveolar bone volume, or even a reversal of degraded alveolar bone [26••, 27•, 28]. It is unclear whether this new bone will be firmly connected to teeth through new Sharpey’s fibers, indicated with red fibers and a question mark. **c** A schematic representation of osteocyte-driven RANKL and sclerostin expression, driving the differentiation of osteoclast precursors into osteoclasts. **d** When both RANKL and sclerostin are inhibited, osteogenic stem cells lining alveolar bone at the periodontal ligament may differentiate into osteoblasts [26••] that produce osteoid, which will turn into bone

During experimental periodontitis, RANKL can be detected at the protein level in osteocytes of alveolar bone, where the percentage of RANKL-expressing osteocytes increases, especially during early stages (days 1–3), in line with a similar induction of osteoclasts. During later stages (days 10 and 20), the percentage of RANKL-expressing osteocytes decreases together with the number of osteoclasts [21]. This upregulation of RANKL in vivo could be under the direct influence of bacterial products such as LPS that may penetrate the periodontium as deep as the alveolar bone containing osteocytes (Fig. 1a). A recent study showed that LPS upregulates RANKL expression in an osteocyte-like cell line, MLO-Y4 [23]. Since the DMP-1 promoter is relatively specific to osteocytes, deletion of RANKL can be targeted specifically in osteocytes, using conditional deletion of floxed RANKL by Cre recombinase. It is important to note that this conditional RANKL model displayed growth retardation and osteopetrosis, indicating a specific role of osteocyte-induced RANKL in bone remodeling [16, 17]. However, unlike the germ-line knockout [18], tooth eruption took place and also the femurs of the osteocyte-conditional knockout displayed normal shaped femurs. Therefore, the role of osteocyte-derived RANKL could be much limited to mechanical stress-induced bone remodeling [29]. Periodontitis was induced in wild-type mice and in mice lacking RANKL only in osteocytes by inoculating a mixture of two periodontitis-associated pathogens, *Pg* and *Fn* three times a week for 2 weeks. Histological analysis revealed that RANKL was induced in alveolar bone osteocytes of wild-type mice and not in alveolar bone osteocytes of the RANKL Cre mice. Strikingly, no periodontitis developed in the osteocyte-specific RANKL-deleted mice, concomitant with no increase in osteoclast number or eroded surfaces. In contrast, wild-type mice exposed to the periodontitis-associated bacteria mixture presented with severe alveolar bone loss, increased osteoclast numbers, and eroded surfaces [24••]. These results clearly demonstrate the pivotal role of RANKL-expressing osteocytes in periodontitis progression. Targeting osteocytes’ RANKL expression locally could therefore be an approach to interfere with periodontitis progression. Since RANKL is upregulated by inflammatory factor tumor necrosis factor-*α* (TNF-*α*), which is upregulated in inflammation-associated diseases such as periodontitis, Kim et al. [30] injected diabetic and non-diabetic rats with infliximab, a TNF-*α* antagonist. Periodontitis was initiated by ligature application to the gums, hereby facilitating a periodontal infection. Rats developed alveolar bone loss, but less so in animals that received infliximab. In parallel, fewer alveolar bone osteocytes were RANKL positive and fewer osteoclasts formed at alveolar bone surfaces [30]. These studies demonstrate a catabolic role for osteocytes: they express RANKL, when modulated with anti-inflammatory drugs; RANKL is diminished and knockout of RANKL specifically in osteocytes even abolished periodontitis.

### Alveolar Bone Osteocytes, Sclerostin, and Periodontitis

The elucidation of the key players of the Wnt-signaling pathway that results in bone formation opens avenues to specifically interfere with bone formation. Importantly, under normal circumstances, alveolar bone osteocytes express the inhibitor of Wnt- signaling, sclerostin, transcribed from the SOST gene [31]. Sclerostin binds to the extracellular domain of LRP5/6 blocking the formation of the LRP/Wnt/Frizzled complex [32], therefore inhibiting the canonical Wnt-signaling pathway, which has a crucial role in the differentiation and activation of osteoblasts [19, 20]. Additionally, sclerostin exerts a catabolic effect on bone via the modulation RANKL/OPG ratio in osteocytes [22, 33], although the specific pathway has yet to be defined. Strategies to neutralize sclerostin, for instance with clinically tested romosozumab, an anti-sclerostin antibody, hold promise for treatment of osteoporosis [25]. Within periodontal tissues, sclerostin is expressed in osteocytes as well as cementocytes [34]. The sclerostin knockout mouse exhibits a mild periodontal phenotype, with no alterations in teeth and a smaller periodontal ligament width due to enlarged cellular cementum [35]. When inducing periodontitis, the sclerostin knockout mice were slightly protected compared to wild-type mice [36]. Knockout of periostin, a structural component of the periodontium, leads to periodontitis due to structural anatomical deviation [37]. These mice were treated with either a viral construct knocking down sclerostin or with a sclerostin neutralizing antibody. Knockdown of sclerostin not only restored alveolar bone height, but also improved the disorganized orientation of the periodontal ligament in these mice [26••]. Similar results were obtained in a periodontitis rat model, using silk sutures around molar to induce periodontitis. These rats developed periodontitis within 4 weeks and after removal of the silk sutures, animals were treated with an antibody against sclerostin. Alveolar bone quality improved and original alveolar loss recovered in the treated group [27•]. Locally administered sclerostin antibody induced limited regeneration of the alveolar bone but this lower effect was attributed to the inability to introduce a sufficient concentration of the antibody. It is theorized that more sophisticated methods of drug delivery to the alveolar bone would increase the efficacy of local treatment in periodontitis [27•]. Independently, Chen et al. showed that treatment of periodontitis-induced rats with sclerostin antibody protected alveolar bone and increased the expression of osteoprotegerin (OPG) [28]. One of the risk factors for using anti-resorptive treatment using either bisphosphonates or anti-RANKL antibody denosumab is osteonecrosis of the jaw [38]. In a study comparing the clinically relevant doses of the sclerostin antibody with bisphosphonates in an experimental periodontitis rat model after ovariectomy, no osteonecrosis developed and also less alveolar bone was lost [39]. Furthermore, all bone anabolic parameters improved after sclerostin antibody treatment [39]. Progressive periodontitis eventually leads to tooth loss, hereby solving the chronic inflammation. Lost teeth can be replaced by implants, which can only be placed when the jawbone has enough height. Liu et al. have administered antibodies to sclerostin in edentulous rats. Rats with extracted teeth that received anti-sclerostin developed a thicker maxillary alveolar ridge height [40]. Since anti-sclerostin improves the periodontal status and RANKL expressed by osteocytes causes periodontitis, it could be that sclerostin influences RANKL and thereby the catabolic nature of osteocytes. This was tested on the MLO-Y-4 cell line that expressed more RANKL when treated with increasing concentrations of sclerostin. Also, sclerostin treated MLO-Y4 cells gave rise to more osteoclasts in osteoclastogenesis assays that were more actively resorbing [22]. Vice versa, osteoclasts may influence the expression of sclerostin in osteocytes. An Opg −/− mouse model was used to study coupling, the relationship between bone formation and bone degradation. Here, conditioned medium from osteoclasts lowered sclerostin expression in osteocytes. Anti-resorptive agents and anti-RANKL-induced sclerostin [33].

### Clinical Relevance of Detecting Sclerostin and RANKL in Crevicular Fluid of Periodontitis Patients

Sclerostin, the osteocyte-expressed and secreted inhibitor of bone formation, can also be found at higher levels in the crevicular fluid from periodontitis patients and could be a more reliable measure for diagnosis or prognosis of disease than RANKL [41]. Also in serum or in gingival biopsies, sclerostin was increased in chronic periodontitis patients [42]. Higher levels of sclerostin were also reported in the peri-implant crevicular fluid in patients with peri-implantitis [43].

## Conclusions

In brief, it can be concluded that osteocytes play a role in periodontitis through the expression of RANKL and sclerostin. These findings are summarized in Fig. 1. Experimental evidence shows that bacterial products such as LPS can enhance RANKL expression of osteocytes. Sclerostin itself acts as positive feedback for RANKL expression. The effect of a bacterial infection on sclerostin expression is still unknown.

It is remarkable that osteocyte-specific RANKL deletion completely blocks periodontitis [24••]. Since no osteoclasts are formed, this may seem logical. But, taking it a bit further, it could suggest a role for osteoclasts in attracting leukocytes to the soft periodontium. It is known that osteoclasts on bone secrete high levels of IL-1β [44], but other leukocyte attracting cytokines could also be secreted by the osteoclast. Therefore, osteocyte-expressed RANKL could well be the beginning of periodontitis progression, through the formation of initial osteoclasts that may attract leukocytes. In recent years, it has become more and more evident that osteoclasts play roles not only as bone degraders [45], but also in preparing the hematopoietic niche [46, 47]. Likewise, it is conceivable that they could be involved in attracting immune cells to the inflammatory environment of the periodontium.

Although hardly considered in clinical periodontitis studies, anti-inflammatory agents such as anti-TNF-α infliximab, used in for instance rheumatoid arthritis, were shown to have a stabilizing effect on the periodontal status of patients [48]. It would be worthwhile in future studies with anti-RANKL denosumab and anti-sclerostin romosozumab [25] to compare periodontal status before and after use of these reagents. Based on our new knowledge on osteocyte-produced proteins and their new role in periodontitis, it would not be surprising when the use of denosumab and romosozumab would have a stabilizing effect on the periodontal status of patients.
